# Supplementary material for: Molecular Sets (MOSES): A Benchmarking Platform for Molecular Generation Models
Source: Front Pharmacol. 2020 Dec 18;11:565644. doi: 10.3389/fphar.2020.565644 (PMC7775580; doi:10.3389/fphar.2020.565644)
Supplement: Supplementary file 1 [file datasheet1.pdf]

frontiers

Supplementary Material

1 MEDICINAL CHEMISTRY FILTERS AND PAINS FILTERS

Medicinal chemistry filters are used to discard compounds containing so-called "structural alerts". Molecules containing such moieties either bear unstable or reactive groups or undergo biotransformations resulting in the formation of toxic metabolites or intermediates.

We filtered the dataset with medicinal chemistry filters (MCFs) that we explain in this section. We used MCFs for rational pre-selection of compounds more appropriate for modern drug design and development. These include some electrophilic alkylating groups, such as Michael acceptors (MCF1-3), alkyl halides (MCF4), epoxide (MCF5), isocyanate (MCF6), aldehyde (MCF7), imine (Schiff base, MCF8), aziridine (MCF9) which are very liable for nucleophilic attack. In many cases, it leads to unselective protein and/or DNA damage. Metabolism of hydrazine (MCF10) furnishes diazene intermediates (MCF11), which are also alkylating warheads. Monosubstituted furans (MCF12) and thiophenes (MCF13) are transformed into reactive intermediates via epoxidation. Their active metabolites irreversibly bind nucleophilic groups and modify proteins. Electrophilic aromatics (e.g. halopyridine, MCF14), oxidized anilines (MCF15) and disulfides (MCF16) are also highly reactive. In vivo, alkylators are trapped and inactivated by the thiol group of glutathione, which is a key natural antioxidant. Azides (MCF17) are highly toxic; compounds containing this functional group particularly cause genotoxicity. Aminals (MCF18) and acetals (MCF19) are frequently unstable and inappropriate in generated structures. In addition, virtual structures containing a large number of halogens (MCF20-22) should be excluded due to increased molecular weight and lipophilicity (insufficient solubility for oral administration), metabolic stability, and toxicity. The detailed mechanism of toxicity for structure alerts mentioned above has been comprehensively described in (Kalgutkar et al., 2005; Kalgutkar and Soglia, 2005).

PAINS (pan-assay interfering compounds) filters are the set of substructure filters proposed to use for reducing the number of false positives, assay artifacts and unspecific bioactive molecules in the screening libraries. It was stated that the presence of certain fragments in a structure could lead to undesirable properties (reactivity, chelation, the formation of colloidal aggregates, dyes) affecting assay results. It should be noted that the analysis of available data from the PubChem database clearly demonstrated the limitations of PAINS filters (Capuzzi et al., 2017; Senger et al., 2016). Indeed, PAINS were observed among the molecules inactive in at least 100 bioassays (the dark chemical matter). Interestingly, structural analysis of well-known drugs revealed PAINS among them. For instance, quinone-based compounds were classified as PAINS, however there are quinone-based drugs approved by the FDA in the market. Despite mentioned above, this approach can be considered as a viable tool for narrowing down the large virtual chemical spaces produced by generative models to drug-like chemical matter.

2 DIVERSE SET OF MOLECULES FROM MOSES

In Figure S1, we show a diverse set of molecules of MOSES dataset. We obtained these molecules by iteratively adding structures with the lowest cosine similarity to the nearest compound in the currently selected set.

3 HYPERPARAMETERS AND TRAINING DETAILS

Character-level recurrent neural networks (CharRNN) used Long Short-Term Memory (Hochreiter and Schmidhuber, 1997) cells stacked into 3 layers with hidden dimension 768 each. We used a dropout

(Srivastava et al., 2014) layer with dropout rate 0.2. Softmax was utilized as an output layer. Training was done with a batch size of 64, using the Adam (Kingma and Lei Ba, 2015) optimizer for 80 epochs with a learning rate of 10^{-3} that halved after each 10 epochs. We display CharRNN model in Figure S2.

Variational autoencoder (VAE) used a bidirectional (Schuster and Paliwal, 1997) Gated Recurrent Unit (GRU) (Cho et al., 2014) with a linear output layer as an encoder. The decoder was a 3-layer GRU of 512 hidden dimensions with intermediate dropout layers with dropout probability 0.2. Training was done with a batch size of 128, utilizing a gradient clipping of 50, KL-term weight linearly increased from 0 to 1 during training. We optimized the model using Adam optimizer with a learning rate of $3 \cdot 10^{-4}$. We trained the model for 100 epochs. We display an autoencoder model in Figure S3.

Adversarial Autoencoders (AAE) consisted of an encoder with a single layer bidirectional LSTM with 512 hidden dimensions, a decoder with a 2-layer LSTM with 512 hidden dimensions and a shared embedding of size 128. The discriminator network was a 2-layer fully connected neural network with 640 and 256 nodes respectively with exponential linear unit (ELU) (Clevert et al., 2016a) activation function (Clevert et al., 2016b). We trained a model with a batch size of 512, with the Adam optimizer using a learning rate of 10^{-3} for 120 epochs. We halved the learning rate after each 20 epochs.

Junction Tree VAE (JT-VAE) We report the experimental results from the official JT-VAE repository (Jin, 2019).

Latent Vector Based Generative Adversarial Network (LatentGAN) pretrained an aused a heteroencoder (Bjerrum and Sattarov, 2018) containing a two-layer bidirectional encoder with 512 LSTM units per layer. Authors added a Gaussian noise with a zero mean standard deviation of 0.1 to the latent codes, resembling VAE with a fixed variance of proposal distributions. The LSTM decoder had 4 layers. The neural network was trained on pairs of randomly chosen non-canonical SMILES strings (Bjerrum, 2017). The autoencoder network was trained for 100 epochs with a batch size of 128 sequences, using Adam optimizer with a learning rate 10^{-3} for first 50 epochs and with an exponential learning rate decay reaching a value of 10^{-6} in the final epoch. LatentGAN uses Wasserstein GAN with gradient penalty (WGAN-GP) (Gulrajani et al., 2017) with a fully connected discriminator with 3 layers of which the first two used the leaky ReLU activation function, and the last layer no activation function. The generator consisted of five fully connected layers with batch normalization and leaky ReLU activation. The GAN was trained for $2{,}000$ epochs using a learning rate of $2 \cdot 10^{-4}$ with Adam parameters $\beta_1 = 0.5, \beta_2 = 0.9$. We display LatentGAN model in Figure S4.

Combinatorial generator randomly joins BRICS fragments. We first cut all molecules from the training set into fragments and compute the frequency of each fragment. We also compute a distribution of the number of fragments in the training set. To produce a molecule, we first randomly sample a total number of fragments that we will use in the molecule. We then iteratively sample fragments according to their frequencies. We omit fragments that will lead to invalid final molecules. For example, if there are currently two free attachment points in the molecule and two fragments left to attach, we cannot attach fragments with more than one attachment points. We also experimented with randomly sampling fragments until there are no more connection points. However, such method performed worse.

N-gram model used 11-gram count statistics with pseudo-count of 0.01. During generation, when there were no statistics available for the current (n-1)-gram, we reduced the context length until some statistics were available. In extreme cases, the context reduced to a single token which is equivalent to a bigram model.

Hidden Markov Model uses Baum-Welch algorithm for training the model. We used HMM with 200 states and trained the model for 100 epochs on a subset of MOSES train set with first 100,000 molecules. Note that HMM uses batch training which leads to high computational costs. To speedup learning, we used K-means|| algorithm (Bahmani et al., 2012) to initialize parameters of the model.

REFERENCES

Bahmani, B., Moseley, B., Vattani, A., Kumar, R., and Vassilvitskii, S. (2012). Scalable k-means+. *Proceedings of the VLDB Endowment* 5

Bjerrum, E. and Sattarov, B. (2018). Improving chemical autoencoder latent space and molecular de novo generation diversity with heteroencoders. *Biomolecules* 8, 131

Bjerrum, E. J. (2017). Smiles enumeration as data augmentation for neural network modeling of molecules. *arXiv preprint arXiv:1703.07076*

Capuzzi, S. J., Muratov, E. N., and Tropsha, A. (2017). Phantom pains: Problems with the utility of alerts for p an-a ssay in terference compound s. *Journal of chemical information and modeling* 57, 417–427

Cho, K., van Merriënboer, B., Gulcehre, C., Bahdanau, D., Bougares, F., Schwenk, H., et al. (2014). Learning phrase representations using RNN encoder–decoder for statistical machine translation. In *Proceedings of the 2014 Conference on Empirical Methods in Natural Language Processing (EMNLP)* (Doha, Qatar: Association for Computational Linguistics), 1724–1734. doi:10.3115/v1/D14-1179

Clevert, D.-A., Unterthiner, T., and Hochreiter, S. (2016a). Fast and accurate deep network learning by exponential linear units (elus). *International Conference on Learning Representations*

Clevert, D.-A., Unterthiner, T., and Hochreiter, S. (2016b). Fast and accurate deep network learning by exponential linear units (elus). *International Conference on Learning Representations*

Gulrajani, I., Ahmed, F., Arjovsky, M., Dumoulin, V., and Courville, A. C. (2017). Improved training of wasserstein gans. In *Advances in neural information processing systems*. 5767–5777

Hochreiter, S. and Schmidhuber, J. (1997). Long short-term memory. *Neural Comput.* 9, 1735–1780

Jin, W. (2019). Accelerated training of junction tree vae. *GitHub*

Kalgutkar, A. S., Gardner, I., Obach, R. S., Shaffer, C. L., Callegari, E., Henne, K. R., et al. (2005). A comprehensive listing of bioactivation pathways of organic functional groups. *Curr. Drug Metab.* 6, 161–225

Kalgutkar, A. S. and Soglia, J. R. (2005). Minimising the potential for metabolic activation in drug discovery. *Expert Opin. Drug Metab. Toxicol.* 1, 91–142

Kingma, D. P. and Lei Ba, J. (2015). Adam: A method of stochastic optimization. *International Conference on Learning Representations* , 1–15

Schuster, M. and Paliwal, K. K. (1997). Bidirectional recurrent neural networks. *IEEE Trans. Signal Process.* 45, 2673–2681

Senger, M. R., Fraga, C. A., Dantas, R. F., and Silva Jr, F. P. (2016). Filtering promiscuous compounds in early drug discovery: is it a good idea? *Drug Discovery Today* 21, 868–872

Srivastava, N., Hinton, G., Krizhevsky, A., Sutskever, I., and Salakhutdinov, R. (2014). Dropout: a simple way to prevent neural networks from overfitting. *The Journal of Machine Learning Research* 15, 1929–1958

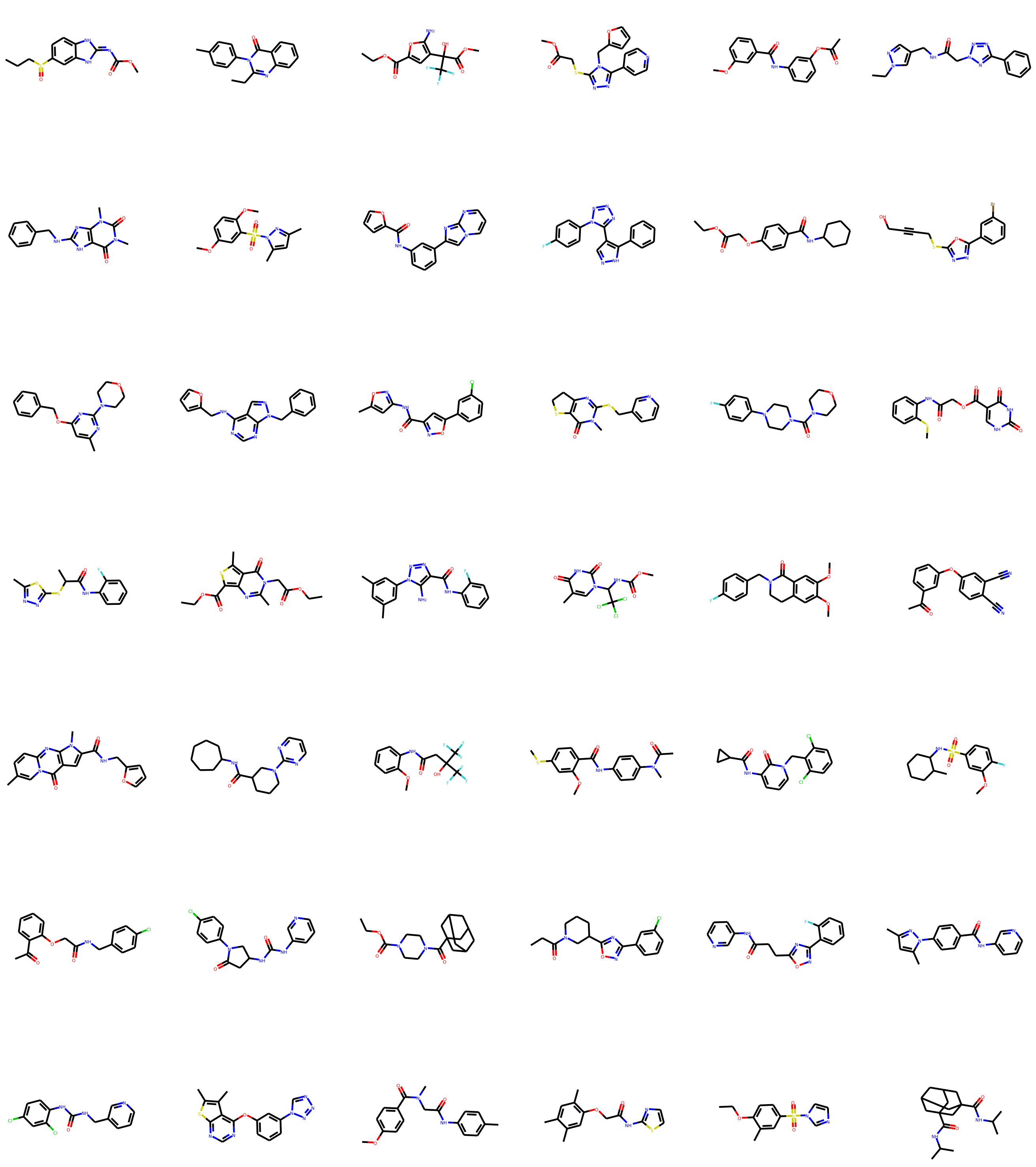

Figure S1. A diverse subset of molecules from MOSES dataset.

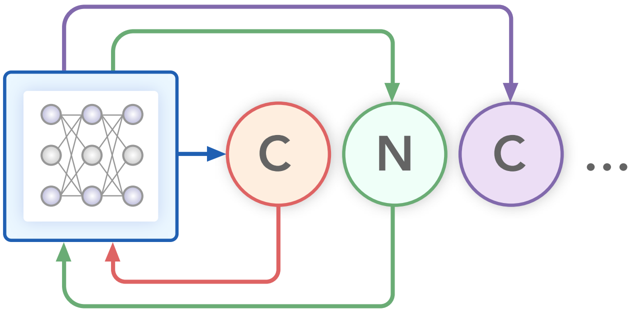

Figure S2. CharRNN model. A model is trained by maximizing the likelihood of known molecules.

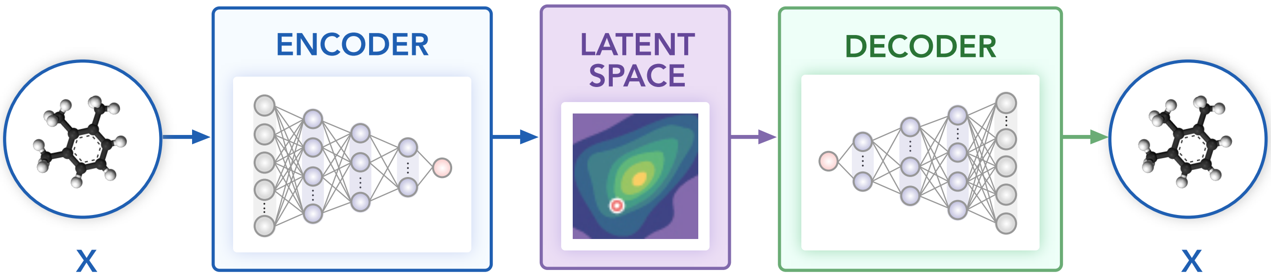

Figure S3. Autoencoder-based models. VAE/AAE forms a specific distribution in the latent space.

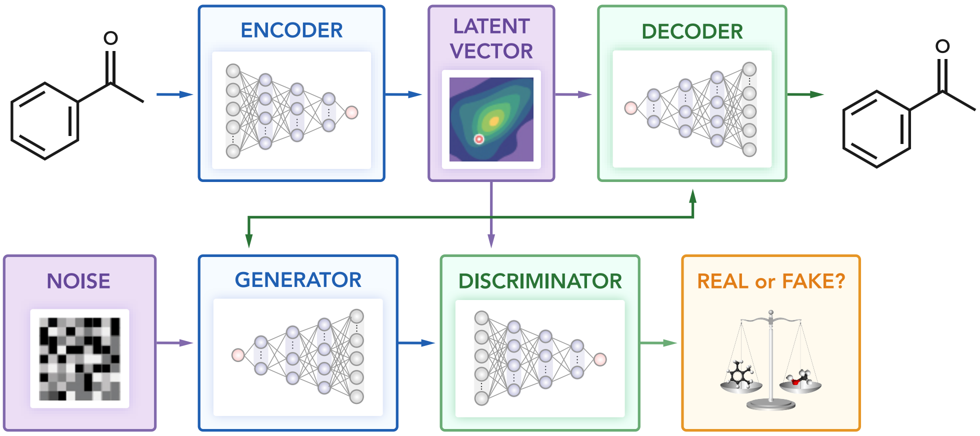

Figure S4. LatentGAN model. A model combines an autoencoder and generative adversarial networks.
